# Chlorhexidine-Loaded Zinc Nanoparticles: A Potent Antibacterial Agent Against *Streptococcus pneumoniae*

**DOI:** 10.1007/s00284-025-04290-2

**Published:** 2025-05-30

**Authors:** Rakesh Kumar, Renu Sharma, Sushila Kaura, Neeraj Sethi, Ikbal Shah, Kumar D. Gahlot

**Affiliations:** 1 Department of Chemistry, Om Sterling Global University, Hisar, India; 2 Department of Pharmaceutical Sciences, Om Sterling Global University, Hisar, India; 3 Department of Biotechnology, Om Sterling Global University, Hisar, India; 4 Department of Microbiology, Om Sterling Global University, Hisar, India; 5 Department of Molecular Biology, Umeå University, Umeå, Sweden; 6 Umeå Centre for Microbial Research (UCMR), Umeå University, Umeå, Sweden

## Abstract

Nanoformulations deliver antibacterial agents synergistically. Positively charged Zn nanocomplexes were used as carriers for chlorhexidine (CHX), developed using ionic liquids. The CHX-loaded Zn nanoparticles (CHZNPs) were characterised through various techniques, including UV–visible Spectroscopy, TEM, FTIR, and Zeta potential analysis. The average diameters of ZNPs and CHZNPs were 27.43 and 29.66 nm, respectively. CHZNPs consistently released CHX, enhancing its antibacterial effect. Tests against antibiotic-resistant *Streptococcus pneumoniae* strain 7465 revealed that CHZNPs significantly reduced bacterial viability. At 100 μg/mL, CHX showed the highest antibacterial activity with the lowest minimal inhibitory concentration (MIC_90_) and minimal bactericidal concentration (MBC_96_) values, followed by CHZNPs, which had lower MIC and MBC values. While ZNPs demonstrated some bactericidal effect at intermediate dosages (12 and 25 μg/mL), they could not fully inhibit bacterial growth. CHZNPs outperformed ZNPs across all concentrations, with an MIC of 40 μg/mL compared to CHX’s 80 μg/mL. ZNPs showed no MIC at tested concentrations. Overall, CHZNPs significantly reduced bacterial viability more effectively than CHX alone, highlighting their potential as a treatment for antibiotic-resistant *S. pneumoniae* infections.

## Introduction

*Streptococcus pneumoniae*, often referred as pneumococcus, is a Gram-positive bacterium that plays a significant role in respiratory infections, particularly pneumonia [1]. It is a leading cause of bacterial pneumonia, sinusitis, and otitis media, contributing to a substantial global burden of disease [2]. This pathogen is encapsulated by a polysaccharide capsule which serves as a major virulence factor, helping the bacterium evade host immune. The transmission of *S. pneumoniae* occurs through respiratory droplets, and it primarily colonises the upper respiratory tract [3]. While many individuals carry the bacterium asymptomatically, it can turn on its virulence under certain conditions, causing infections that range in severity from mild to life threatening [4–6]. Pneumococcal diseases are more prevalent in young children, the elderly, and individuals with weakened immune systems [7].

Antibiotic resistance in *S. pneumoniae* poses a significant public health challenge, complicating the treatment of infections caused by this bacterium [8]. Over the years, the widespread use and sometimes misuse of antibiotics have exerted selective pressure on bacterial populations, fostering the emergence of resistant strains [9, 10], *S. pneumoniae*, responsible for a range of respiratory infection [11] has shown a remarkable ability to develop resistance to multiple classes of antibiotics [12]. One key factor contributing to antibiotic resistance in *S. pneumoniae* is the horizontal transfer of resistance genes, facilitated by the bacterium’s capacity for genetic recombination [13]. This enables the exchange of genetic material between different strains, leading to the acquisition of resistance traits [1]. The emergence of penicillin-resistant strains was a notable early development, prompting adjustments in the treatment guidelines. The introduction of pneumococcal conjugate vaccines has played a crucial role in reducing the incidence of invasive pneumococcal diseases [14]. However, the selective pressure exerted by antibiotic use remains a concern. Resistance to macrolides, such as Erythromycin, has also been documented, which further limiting treatment options [15]. Monitoring and surveillance of antibiotic resistance pattern in *S. pneumoniae* are essential for guiding therapeutic strategies and public health interventions [16].

The utilisation of chlorhexidine-loaded Zinc nanomaterials (CHZNPs) presents a promising avenue for addressing antibiotic resistance in *S. pneumoniae*. Chlorhexidine is an antiseptic with broad-spectrum activity against various microorganisms, including bacteria [17]. When incorporated into metallic nanomaterials, such as nanoparticles, it can enhance its efficacy and provide a multifaceted approach to combat antibiotic-resistant strains of *S. pneumoniae* [18]. Metallic nanomaterials, such as silver, zinc, or copper nanoparticles, have inherent antimicrobial properties, making them effective agents against a range of bacteria, including antibiotic-resistant strains [19]. When combined with chlorhexidine, the synergistic effect may lead to increased antibacterial activity, potentially overcoming resistance mechanisms exhibited by *S. pneumoniae*. The nanomaterials can serve as carriers, facilitating controlled and sustained release of chlorhexidine, and optimising its therapeutic effects [20].

The nanoscale dimensions of these materials offer advantages in terms of increased surface area and enhanced penetration into the bacterial cells, allowing for more efficient interaction with the target pathogen. This targeted delivery system may minimise the potential side effects associated with systemic administration of antimicrobial agents [21]. Furthermore, the use of chlorhexidine-loaded metallic nanomaterials aligns with the principles of precision medicine, enabling tailored therapeutic approaches that specifically target the resistant strains of *S. pneumoniae* [22]. Investigations in this field are essential to optimise the formulations, dosage, and delivery mechanisms, ensuring both efficacy and safety in clinical applications. Overall, the development of such nanomaterial-based strategies represents a cutting-edge approach in the ongoing battle against antibiotic resistance in *S. pneumoniae*. Here, in this study, we have designed, characterised, and tested antibacterial activity of CHZNPs against antibiotic-resistant *S. pneumoniae* strain 7465. The antibacterial effects of CHZNPs were found much better than ZNPs, indicating the strong antibacterial attributes of the designed Nanoformulations.

## Materials and Methods

### Chemicals, Compounds and Bacterium

The chlorhexidine and 1-dodecyl-3-methylimidazolium iodide were procured from MP Biomedicals, LLC, India. The compound Zn (NO_3_)_2_ was purchased from Sigma Aldrich, an establishment situated in India. Hi-media Laboratories Pvt. Ltd., Mumbai, India, supplied the culture media, penicillin, and streptomycin. Gram-positive, antibiotic-resistant bacterial strain, *S. pneumoniae* 7465 was kindly provided by the CSIR-IMTECH, Chandigarh, India. The chemicals utilised in this study were of analytical grade.

### Synthesis of Cationic Zinc Nanoparticles (ZNPs)

To create the positively charged NPs, a 1.0 mL solution of 0.1 M Zn (NO_3_)_2_ was combined and agitated with 6.2 mM 1-dodecyl-3-methylimidazolium iodide. The 0.4 M NaBH_4_ aqueous solution, which had been prepared in advance, was then rapidly added drop by drop to the agitated solutions until a golden colour appeared. Subsequently, to eliminate the residual ionic liquids, the colloidal solutions were subjected to centrifugation (8000 rpm) for approximately 20 min [23].

### Synthesis of Chlorohexidine-Loaded Cationic Zinc Nanoparticles (CHZNPs)

A 1.5 mg/mL alcoholic solution of chlorohexidine (CHX) was produced and added to the alcoholic suspension of Zn (NO_3_)_2_ (2 mg/mL). The mixture was agitated for 24 h at room temperature using a magnetic stirrer. Subsequently, the suspension was subjected to drying at a temperature of 50 °C in an oven. The resulting dried product was then stored in a light-protected container within a refrigerator to conduct antibacterial investigations. Before conducting the antimicrobial experiments, the powder was dissolved in sterile distilled water [24].

### Optimization of CHZNPs Synthesis

To enhance the synthesis of Chlorohexidine-loaded Cationic Zinc Nanoparticles (CHZNPs), an optimisation study was performed to evaluate the impact of various parameters, including the concentrations of chlorohexidine (CHX) and zinc nitrate [Zn(NO_3_)_2_], stirring conditions, drying temperature, and storage conditions. The concentration of CHX was varied from 0.5 to 2 mg/mL, and the zinc nitrate concentration was tested at 1, 2, and 3 mg/mL to determine the optimal ratio for effective drug loading. Stirring conditions were optimised by testing different stirring speeds (200, 400, and 600 rpm) and stirring times (6, 12, and 24 h) to enhance the interaction between CHX and the nanoparticles. The drying temperature was set at 40, 50, and 60 °C for varying durations (6, 12, and 24 h) to identify the best conditions for drying and maintaining the stability of the nanoparticles.

The resulting formulations were stored at room temperature and 4 °C to assess their stability over time. The optimization study demonstrated that the highest drug loading efficiency was achieved under specific conditions, with CHZNPs exhibiting an initial burst release of CHX followed by sustained release over 24–48 h. Antimicrobial activity tests showed that the optimised CHZNPs formulation had significantly lower MIC90 and MBC96 values against *S. pneumoniae* compared to free CHX and zinc nanoparticles, confirming the enhanced antibacterial properties of the optimised CHZNPs.

### Biophysical Characterisation of Synthesised ZNPs and CHZNPs

Various analytical tools were employed to characterise the synthesised nanoparticles. To validate the synthesis of designed nanoparticles, widely used UV–Visible spectroscopy [25], was employed. Distilled water was used as a benchmark control. The absorbance spectra of the colloidal sample were obtained using a UV-1800 UV–Visible spectrometer manufactured by Shimadzu Corporation in Kyoto, Japan. The spectrum was measured in the wavelength range of 200–800 nm [13].

To ascertain the morphology, shape, and size of the designed formulations, the Transmission Electron Microscopy (TEM) [26] was used. The TEM measurements were conducted with a HITACHI H-800 microscope operating at 200 kV. To prepare the TEM grid, a small amount of the diluted solution was deposited onto a copper grid that had been covered with a layer of carbon. The solution was then dried using a lamp [27].

The stability index of the designed formulations was monitored by Fourier-Transform Infrared Spectroscopy (FTIR spectroscopy) to categorise the substances that are responsible for both stabilising the nanoparticles and reducing the metal contents [28]. The Vertex 70 (Bruker, Germany) spectrometer, operating in the wavelength range of 400–4000 cm^−1^, was employed to analyse the functional group at the surface of designed formulations.

The zeta potentials of the designed nanoparticles were determined using a Zetasizer (Nano ZS-90, Malvern Instruments, UK) [29]. A total volume of 700 µL of Zn (NO_3_)_2_ suspensions was prepared and examined at 25 °C with a scattering angle of 90°.

### Loading Efficiency of CHX

The loading efficiency of chlorohexidine (CHX) on ZNPs was assessed using UV/VIS spectroscopy [30] at a wavelength of 254 nm. The unloaded CHX amount was computed after the loading method, and the CHX loading percentage (%) was determined using the following formula:

$${\text{Loading efficiency }}\% = \frac{{{\text{Total concentration of CHX}} - {\text{Concentration of unloaded CHX}}}}{{\text{Concentration of unloaded CHX}}}$$

### In Vitro Release Profile of CHZNPs

To investigate the release profile of designed CHZNPs, the dialysis sac method [31] was employed. A total of 10 mg of CHZNPs were placed into a dialysis sac. The sac was then submerged in a solution consisting of 10 mL of distilled water, 25% ethanol, and 0.1 M phosphate buffer saline at a pH of 7.4. The solution was continuously swirled at a rate of 100 rpm, while maintaining a constant temperature of 37 °C. At regular intervals of 1, 2, 3, 6, 12, and 24 h, 1 mL sample was harvested and analysed using High-Performance Liquid Chromatography (HPLC) with an Agilent 1200 Infinity Series instrument [32]. The analysis was performed using a ZORBAX SB C-18 column (5 μm, 150 × 4.6 mm) and a mobile phase consisting of Acetonitrile:Water (80:20 v/v). The compound of interest, Chlorohexidine, was detected at a wavelength of 254 nm, with 6.15 min retention time.

### Determination of Antibacterial Activity of Designed CHZNPs

The antibacterial attributes of the designed CHZNPs, the minimum inhibitory concentration (MIC), and the minimum bactericidal concentration (MBC) were determined against *S. pneumoniae* strain 7465 using the microdilution broth method [33], with three biological replicates. The solutions that were examined included a 0.2% concentration of CHX, a combination of ZNPs and CHZNPs. The tests were performed by the guidelines set by the Clinical and Laboratory Standards Institute (CLSI). To determine the MIC, twofold serial dilutions (up to seven times) of the experimental solution was prepared in 96-well microtiter plates. Mueller Hinton broth (MHB) was used as negative control. To achieve a volume of 90 μL, *S. pneumoniae* was cultivated in Brain Heart Infusion (BHI) broth and the turbidity was adjusted to a range of 0.10–0.14 at an optical density (OD) of 600 nm. This resulted in a concentration of 1.6 × 10^6^ colony forming units per millilitre (CFU/mL) of bacteria. Subsequently, the suspension was diluted at a ratio of 1-part suspension to 20 parts broth culture. A volume of 10 μL of *S. pneumoniae* suspension was introduced into each microtiter plate, and plates were incubated at 37 °C for 24 h. Following incubation, the OD_600_ nm was recorded using a microplate reader (Biotek Micro Plate Reader).

The positive control group was composed of culture media inoculated with *S. pneumoniae*, while the negative control group consisted of culture media devoid of *S. pneumoniae.* The MIC value was determined as the minimum concentration of each experimental solution that inhibited 90% of *S. pneumoniae* growth, in comparison to the negative control group. To measure the MBC of the designed formulations, an experimental solution with concentration equivalent to or higher than that of MIC was placed was placed on Tryptic soy Agar plates. These plates were incubated at 37 °C, and bacterial colonies were enumerated after 24 h incubation. The MBC was determined as the lowest concentration at which the bacteria in the initial inoculum were nearly completely eradicated. This occurred when fewer than four discernible colonies were visible on the agar plates following 24 h incubation at 37 °C.

## Results

### Optimization Studies

The optimization study for Chlorohexidine-loaded Cationic Zinc Nanoparticles (CHZNPs) revealed the following key results: The highest drug loading efficiency was achieved with a CHX concentration of 1.5 mg/mL and a Zn(NO_3_)_2_ concentration of 2 mg/mL, which are the concentrations used in the synthesis. The stirring speed was set to 400 rpm for 24 h, which was found to be optimal for effective drug loading. Drying at 50 °C for 12 h ensured the stability of the nanoparticles. The in vitro release profile showed that 95% of pure CHX was released in 4 h, while CHZNPs released 80% CHX in 24 h, demonstrating a sustained release of the drug. The MIC90 for CHZNPs was 50 μg/mL, and the MBC96 was similarly observed at the same concentration, confirming the enhanced antimicrobial activity against *S. pneumoniae* compared to free CHX. Storage at 4 °C provided the best stability for the formulation.

### Biophysical Characterisation of CHZNPs Nanoformulation

#### Particle Size and Zeta Potential

**Fig. 1:**
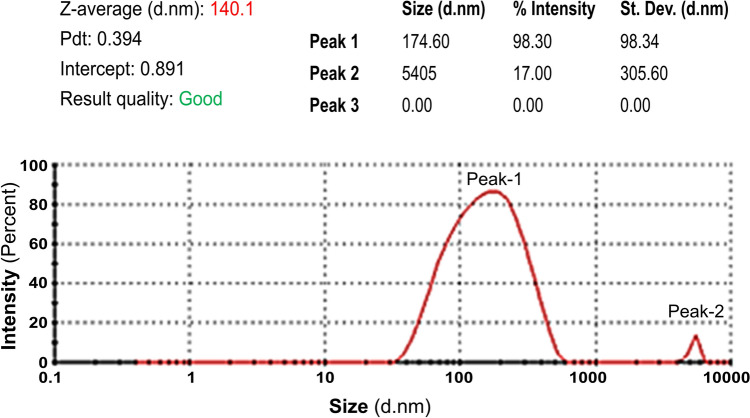
Particle size analysis of chlorhexidine (CHX) carrying Zn-nanoparticles, the CHZNPs nanoformulation. The size of CHZNPs (red line) was predominantly found to be 140.1 d.nm (Peak-1) with an intensity of 98.30%. The Master sizer 3000 + serves as a dependable companion for conducting particle size analysis

**Fig. 2:**
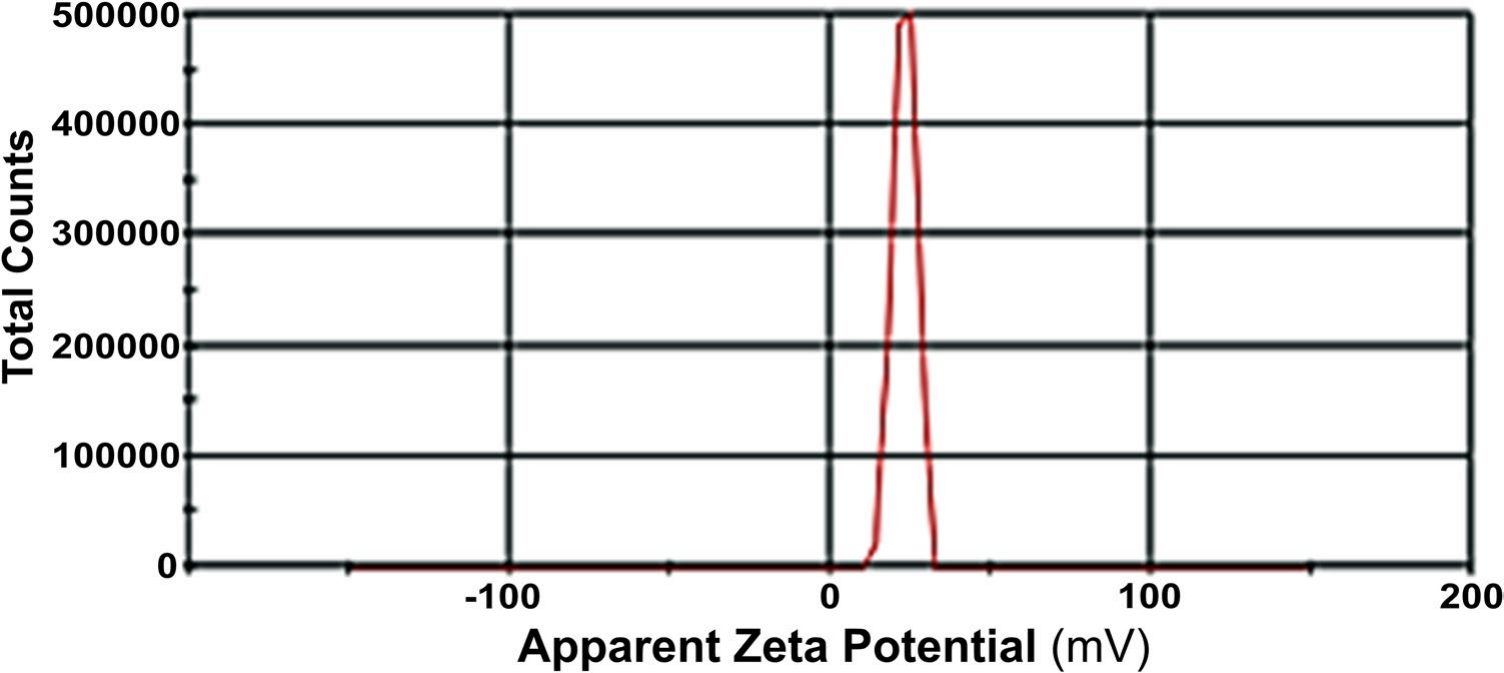
Zeta Potential of CHX carrying CHZNPs nanoformulation. The Zeta potential of the designed nanoparticles was determined using a Zetasizer (Nano ZS-90, Malvern Instruments, UK). The Zeta potential of CHZNPs (red peak) was measured to be + 23 and + 45.3 mV

A comprehensive examination was performed on the ZNPs and CHZNPs to ascertain their respective particle size and Zeta potential. The dimensions of the ZNPs and CHZNPs were found to be 124 and 140.1 nm, respectively, as shown in Fig. 1. The Zeta potentials were measured to be + 23 and + 45.3 mV, as depicted in Fig. 2.

#### Per Cent Encapsulation Efficiency

The effectiveness of encapsulation depends on the molecule of interest, technique used to encapsulate materials, and media employed in the synthesis of nanoparticles [34]. The encapsulation effectiveness percentage of CHX was measured by analysing the supernatant using HPLC to quantify the quantity of unbound material. The data indicated that the encapsulation effectiveness of CHX was 70.8%. Both CHX and CHZNPs exhibit hydrophobic characteristics. They exhibit exceptional solubility in a gum solution derived from n-butanol. Consequently, the CHX was encapsulated more efficiently in nanoparticles due to this affinity.

### Morphological Characterisation of ZNPs and CHZNPs by TEM

**Fig. 3:**
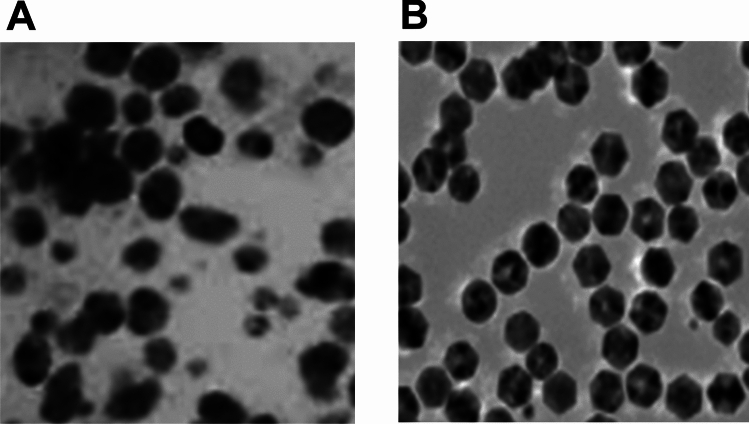
Transmission Electron Microscopy (TEM) analysis of Zn-nanoparticles (**A**) and CHX carrying CHZNPs nanoformulation (**B**). The TEM measurements were conducted with a HITACHI H-800 microscope operating at 200 kV. To prepare the TEM grid, a small amount of the diluted solution was deposited on a copper grid that had been covered with a layer of carbon. The solution was then dried using a lamp. The dimensions of Zn nanoparticles, ZNPs (A) and CHX carrying CHZNPs nanoformulation (B) were varied between 54 and 68 nm (for ZNPs) and 75–84 nm (for CHZNPs), respectively

The ZNPs and CHZNPs were isolated and exhibited a uniform spherical morphology, as evidenced by TEM (Fig. 3). Their dimensions were varied between 54 and 68 nm for ZNPs and 75–84 nm for CHZNPs.

### FTIR Analysis of Drug Samples

**Fig. 4:**
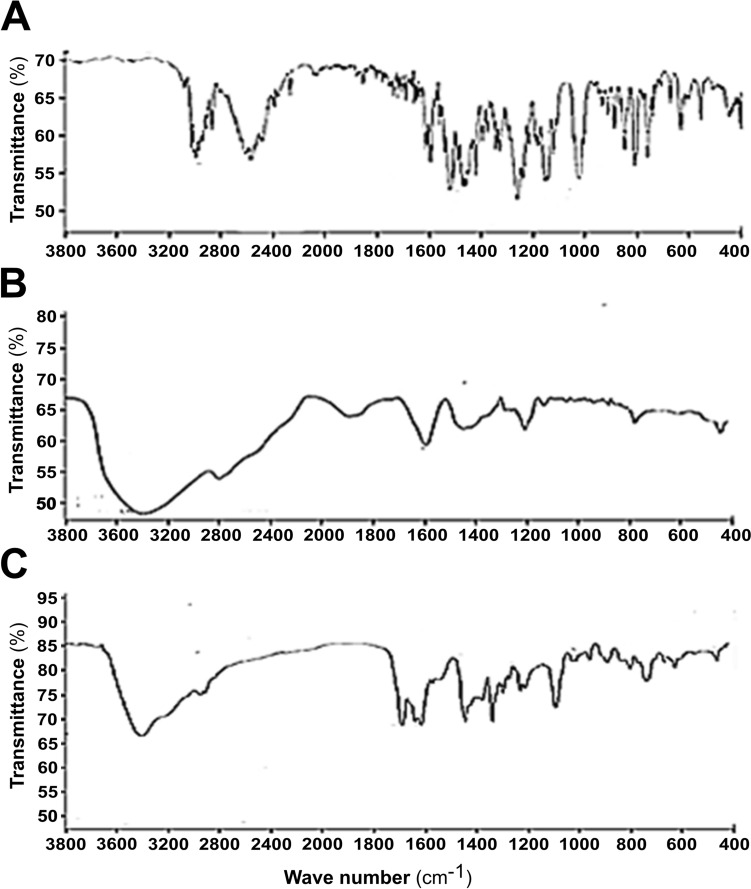
Fourier-Transform Infrared (FTIR) spectroscopy analysis of chlorhexidine, CHX (**A**), Zn-nanoparticles, ZNPs (**B**), and CHX carrying CHZNPs nanoformulation (**C**). The Vertex 70 (Bruker, Germany) spectrometer, operating in the wavelength range of 400–4000 cm^−1^ was employed to analyse the functional group at the surface of designed formulations. ZNP peaks appeared at 3398, 2899, and 1612 cm^−1^ in the spectral graphs. The imidazolium groups’ surface amide and hydroxy groups’ N–H and O–H bonds are visible at 3398 cm^−1^ in the ZNPs sample (B). The cationic aliphatic side chain’s stretching of aliphatic CH bonds causes vibrations at 2899 cm^−1^. A peak at 1612 cm^−1^ supports ZNPs’ acrylic carbonyl group; CHZNPs spectral graph (C) show this peak

The functional groups responsible for stabilising, capping, surfacing, and reducing ZNPs were verified using FTIR analysis (Fig. 4). The graph displays distinct peaks at 3398, 2899, and 1612 cm^−1^, which are indicative of ZNPs. The signal seen at 3398 cm^−1^ in the ZNPs sample corresponds to the N–H bond of the amide and the O–H bond of the hydroxy groups, which are present on the surface imidazolium groups. In addition, the vibrations occurring at around 2899 cm^−1^ can be attributed to the stretching of aliphatic CH bonds present in the cationic aliphatic side chain. The occurrence of a peak at around 1612 cm^−1^ further substantiates the existence of an acrylic carbonyl group in ZNPs. This peak also appears in the graph of CHZNPs (Fig. 4).

### In Vitro Drug Release of CHX and CHZNPs

**Fig. 5:**
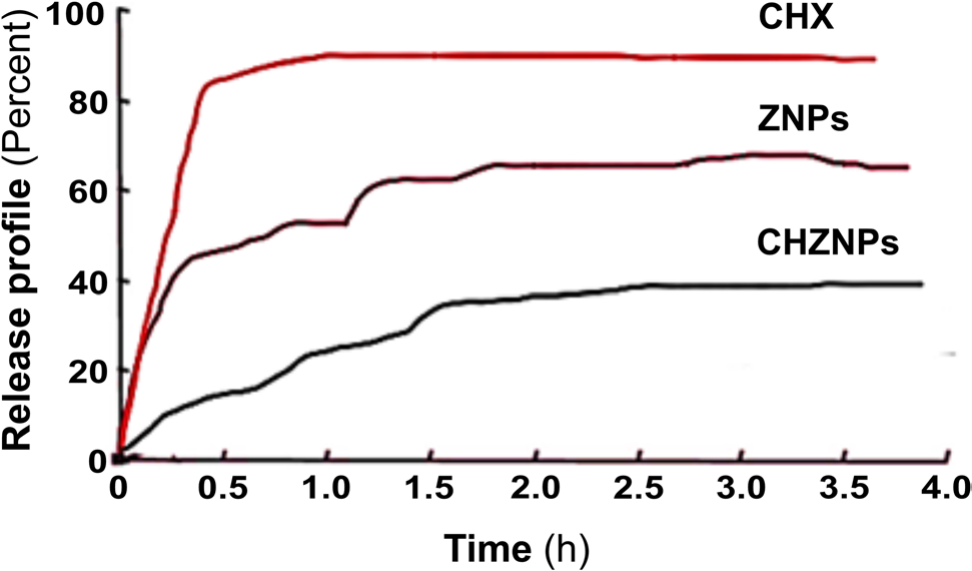
In vitro release profile of chlorhexidine (CHX), Zn-nanoparticles (ZNPs) and CHX carrying Zn-nanoparticles (CHZNPs) nanoformulation. Dialysis sacs were used to examine anticipated CHZNPs release profile. The dialysis bag contained 10 mg CHZNPs. The sac was then submerged in 10 mL distilled water, 25% ethanol, and 0.1 M pH 7.4 phosphate buffer saline. The solution stayed at 37 °C by swirling at 100 rpm. High-performance liquid chromatography (HPLC) (Agilent 1200 Infinity Series) was used to analyse 1 mL samples at 1, 2, 3, 6, 12, and 24 h. Analyses were performed on a 5 μm ZORBAX SB C-18 column (150 × 4.6 mm) using an 80:20 v/v Acetonitrile: Water mobile phase. With 6.15 min retention, chlorohexidine (CHX) was detected at 254 nm. Encapsulation made CHZNPs release more sustainably and 95% of pure CHX was released in 4 h. One-hour post-treatment, CHZNPs release 20% CHX. In 24 h, CHZNPs released 80% CHX. Slow CHX emission from CHZNPs ensures sustainability

Continuous nanoparticle-mediated bioactive chemical release protects against rapid metabolism and degradation [35]. The in vitro release profile of CHX and CHZNPs is shown in Fig. 5. It was found that within 4 h, 95% of pure CHX was released and because of encapsulation, CHZNPs release was more sustainable. One hour after treatment, CHZNPs release 20% CHX. Within 24 h, CHZNPs released 80% CHX. Slow release of CHX from CHZNPs, ensuring their more sustainable release.

### Antimicrobial Property of the CHX and CHZNPs

**Fig. 6:**
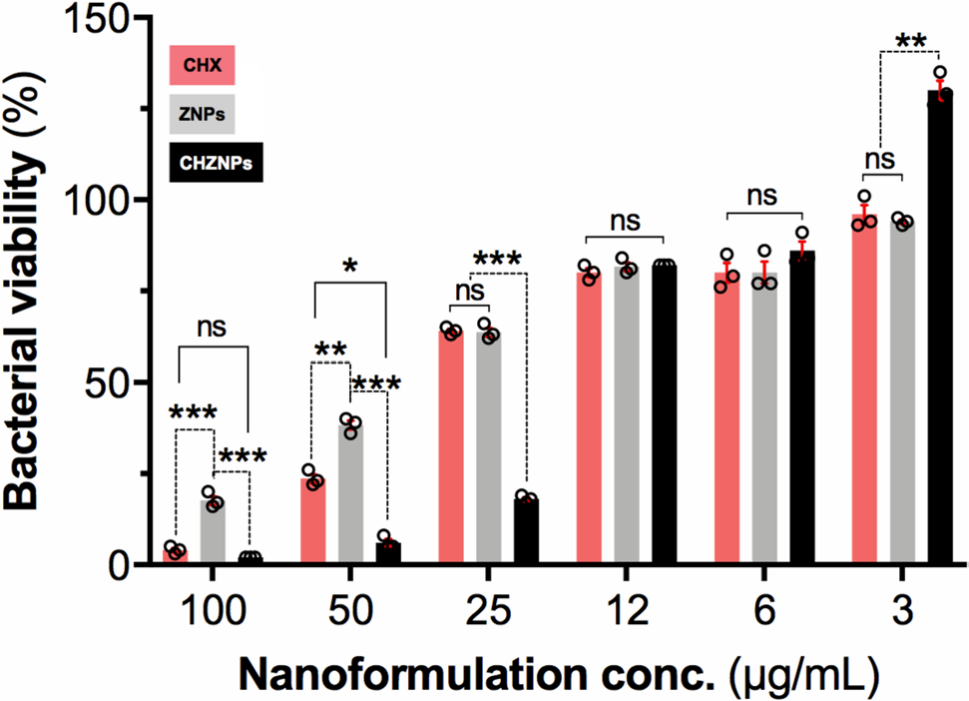
Viability of *Streptococcus pneumoniae* strain 7465 when treated with different concentrations of designed nanoformulations. The survival percentage (%) of *S. pneumoniae* strain 7465 was determined upon treating with a range of indicated concentrations of chlorhexidine (CHX), Zn-nanoparticles (ZNPs) and designed CHX carrying Zn-nanoparticles (CHZNPs) nanoformulation. The survival % inferred from the minimum inhibitory concentration (MIC_90_) and minimum bactericidal concentration (MBC_96_), using Microdilution broth method with CSLI guidelines. The CHZNPs nanoformulation at 50 μg/mL had the lowest both MIC_90_ and MBC_96_ values, followed by CHX at 100 μg/mL and ZNPs with an unknown value. ZNPs alone did not inhibit 90% of *S. pneumoniae* growth and the MBC_96_ and MIC_90_ values of CHZNPs were unknown. At a concentration of 25, 50 and 100 μg/mL CHZNPs outperformed to both CHX and ZNPs while at a concentration of 6 and 12 μg/mL no significant difference was observed among CHX, ZNPs and CHZNPs. At a concentration of 3 μg/mL, the CHZNPs favoured *S. pneumoniae* viability in comparison to both CHX and ZNPs. Bars represent the mean (±) of standard deviations of the survival % of *S. pneumoniae* from three biological replicates. GrphPad Prism-7.0a (Mac OS X version; San Diego, CA, USA) was used for statistical analysis. The statistical significance among CHX, ZNPs and CHZNPs was determined using One-Way ANOVA with Tukey’s multiple comparisons test, with a single pooled variance. The difference in variance with a *p*-value of < 0.05 was considered significant. The *p*-values are indicated by *P < 0.05, **P < 0.01, ***P < 0.001 and > 0.05 (ns; non-significant)

As per broth microdilution test, all three experimental solutions effectively suppressed the growth of *S. pneumoniae* when compared to the control group. The MIC_90_ values of all the tested solutions were comparable to their MBC_96_ values. The CHZNPs solution at a concentration of 50 μg/mL had the lowest MIC_90_ and MBC_96_ values, followed by CHX at a concentration of 100 μg/mL, and ZNPs with an unspecified value. The initial concentration of ZNPs was insufficient to achieve 90% inhibition of *S. pneumoniae.* Therefore, it was not possible to obtain the precise MBC_96_ and MIC_90_ values for CHZNPs. The results revealed that except for the concentration of 80 μg/mL, CHZNPs exhibited a significant decrease in bacterial viability compared to CHX. However, ZNPs had a comparable antibacterial impact to CHX only at moderate concentrations (10 and 20 μg/mL), and their effects were significantly inferior to those of CHX at all other concentrations (3, 6, 40, and 80 μg/mL). The effects of CHZNPs were significantly superior to those of ZNPs at all tested concentrations (Fig. 6).

## Discussion

Nanoparticles have wide applications in medical field, but more recent interest has arisen primarily in the response to emerging field of drug delivery systems, where it is being used as nanocarriers. At present, many drugs fail in clinical trials because the compound cannot be delivered to the site where it is needed without having some interaction with the human body. The result can range from triggering a severe immune response to toxic side effects. Getting the active drug/compound to where it is required, and effectively delivering it are one of the holy grails in the treatment of diseases, ranging from inflammations to bacterial infections.

The global pharmaceutical industry has been developing compounds for over a century, but many of these are not so effective. Considering this objective in mind, we have developed Chlorohexidine-loaded Zinc nanoparticles (CHZNPs) and monitored their antibacterial attributes on Gram-positive, antibiotic-resistant bacterial strain, *S. pneumoniae* 7465.

Customising the size, shape, and/or surface chemistry of nanoparticles enables them to fulfil various needs [36]. The intended action of nanoparticles depends on their target organ or tissue. Both tumour permeability and efficient cellular absorption of nanoparticles, which are dependent on their size, are prerequisites for nanotherapeutics [37]. Furthermore, influencing the ideal size of a nanoparticle is the target site and the kind of targeted tissue [38]. Many size-dependent phenomena including chemical, electrical, magnetic, and mechanical characteristics are introduced by the high surface-to-volume ratio of nanoparticles combined with size effects (quantum effects). Since particle size is so important to nanoparticle properties, particle size is a necessary step in characterising nanoparticle qualities [39]. The obtained particle size of CHZNPs ranging from 124 to 140.1 nm in our study suggests a stable nano-formulation.

The zeta potential (ζ) is a surface charge measurement and the scientific term for electrokinetic potential in colloidal systems [40]. Particle stability as well as cellular absorption and intracellular trafficking can be affected. An electrical double layer and van der Waals forces working in balance determine the stability of a colloidal system where Zeta potential of nanocarriers is crucial. It provides data on particle charge and particle tendency in a formulation to aggregate or stay discrete and is obtained using a Zetasizer or other methods [41]. In general, particles that have Zeta potential greater than + 30 mV or greater than − 30 mV are regarded to be stable [42]. The attracting forces cause the emulsion droplets or colloids to gather if the Zeta potential drops below a specific threshold. On the other hand, a stable system is maintained by a high Zeta potential (either positive or negative), usually more than 30 mV [43]. While repulsion between particles with identical electric charges prevents particle aggregation and assurances easy redispersion, extremely positive or negative Zeta potential values produce stronger repulsive forces [44]. In our study, Zeta potential of Chlorohexidine (CHX) and CHZNPs was measured to be + 23 and + 45.3 mV, respectively, demonstrating that the generated nanoformulations were rather stable. We found that CHX was encapsulated in the CHZNPs quite effectively with a 70.8% efficacy. The encapsulation efficiency percentage of a nanoparticle indicates how much of a nanoformulation is effectively encapsulated [45]. Its computation is the total drug added less the free, non-entrapped drug multiplied by 100. Moreover, important is the drug release time course from nanoparticles, which establishes the quantity of free drug available over time [46]. The toxicity profile of the formulation may either be altered, or it could have a therapeutic benefit by free drug. Previous studies indicated that the percentage of energy efficiency increased from 78.63 ± 4.2% to 87.93 ± 11.4% in direct proportion to the increase in CHX accumulation during PLGA/CHX formulation preparation and an encapsulation efficiency of 78.63 ± 4.2% led to a drug loading of 10.49 ± 1.37%, which dramatically increased to a high of 19.26 ± 7.42% when more CHX was added, resulting in a ratio of 125:50 [47].

TEM analysis provides valuable insights into various aspects of the sample, including sample size, particle size statistics, crystal grain size, crystal structure, crystal phase composition, and atomic arrangement order [48]. The size of nanoparticles influences their release rate. TEM measures the size of the particles in a dehydrated and isolated atmosphere, resulting in a decrease in particle size [49]. The TEM analysis determined the respective size of ZNPs and CHZNPs ranged from 54 to 68 nm and 75 to 84 nm, indicating stable nano-formulations. Nanoparticles are distributed throughout various organs in the body based on their specific structure and size. The form and size of nanoparticles have a crucial role in determining their longevity, compatibility with living organisms, and capacity to penetrate biological tissue. Nanoparticles of smaller sizes exhibit increased durability in the bloodstream compared to nanoparticles of larger sizes. In a previous study, TEM analysis indicated that the average size of AgNPs + and CHX@AgNPs + nanoformulations was roughly the same [50]. Respective mean diameters of AgNPs + and CHX@AgNPs + was determined to be of 27.43 and 29.66 nm.

The FTIR analysis of the CHZNPs suggests that the designed nanoformulations were stable. FTIR spectroscopy is a versatile technique, to measure a wide range of frequencies [51]. The use of this technique encompasses several methods for examining nanomaterials, such as discerning their specific type and composition, ascertaining their level of purity and quality, tracking the progress of their synthesis and processing, evaluating the features of their surface and interface, and investigating the interactions and impacts of these nanoparticles [52]. FTIR spectroscopy quantifies molecular vibrations and rotations induced by infrared radiation of a particular wavelength. It can detect alterations in functional groups inside biomolecules and ascertain the chemical makeup of the surface of a nanoparticle. FTIR spectroscopy can be employed to observe and analyse chemical processes occurring on the surface of nanoparticles, considering different factors including temperature and the surrounding gaseous conditions [53]. In previous studies, Free/MNP@CHX immobilised FTIR, the Aminosilane shell surrounding the magnetic core displayed a large band at 3355 and 3393 cm^−1^ in both samples, indicating N–H stretching and bending vibrations. Moderate signals are produced by NH wagging at 868 cm^−1^ for CHX and 847 cm^−1^ for MNP@CHX. In biguanide derivatives, C=N stretching vibrations may produce adsorption bonds at 1600 cm^−1^ [54]. In our study, ZNP peaks appear at 3398, 2899, and 1612 cm^−1^. The imidazolium groups’ surface amide and hydroxy groups’ N–H and O–H bonds are visible at 3398 cm^–1^ in the ZNPs sample. The cationic aliphatic side chain’s aliphatic CH bands stretch, causing vibrations at 2899 cm^−1^. A peak at 1612 cm^−1^ supports ZNPs’ acrylic carbonyl group.

Evaluation of in vitro drug release is an essential parameter to evaluate the quality, safety, and effectiveness of any drug delivery systems that utilise nanoparticles [55]. Many instruments are utilised to quantify the accessibility of drugs during the initial phases of product development, ensuring quality control for batch release, evaluate factors related to formulation, assess manufacturing methods that impact bioavailability [56], which determine the pharmaceutical quality of a product, and monitor formulation design and batch-to-batch variation. Here, we found that within 24 h, CHZNPs released 80% CHX indicating a sustainable release of nanoparticles.

Nanoparticles exhibit antibacterial and biocidal characteristics, allowing them to effectively battle bacteria, fungi, and viruses [57]. Here, we found that the antibacterial effect of the designed nano-formulation, CHZNPs on *S. pneumoniae* 7465, was much higher than that of ZNPs alone at all tested concentrations, indicating which CHZNPs hold strong antibacterial property.

The constant release of CHX from CHZNPs, made our nanoformulations more effective antibacterial agent at least for the *S. pneumoniae* 7465. Nanoparticles exhibit properties that make them very appropriate as antimicrobial agents, including a large surface area, charge, ability to deliver a big amount of antibiotics or other compounds, size, and shape [58]. Previous study demonstrated that CHX@AgNPs + nanoformulations had a much greater antibacterial impact, reducing the bacterial viability as compared to CHX [59]. The AgNPs + exhibited comparable antibacterial properties to CHX only at moderate doses (12–25 μg/mL), whereas their effectiveness was much lower than that of CHX at other concentrations (3, 6, 50, and 100 μg/mL). The MIC values of CHX@AgNPs + and CHX were found to be 50 and 100 μg/mL, respectively [60].

Considerable efforts have been made to assess the antiseptic efficacy of new nanoformulations carrying CHX as main antimicrobial agent. Based on the current situation and the results obtained from our in vitro study, we accentuate the need for additional research on the use of these CHX-loaded nanoformulations. In summary, here we created a CHX carrying CHZNPs nanoformulation that has substantial antibacterial effect against the Gram-positive, antibiotic-resistant bacterium, *S. pneumoniae* 7465.

An economic analysis of Chlorohexidine-loaded Cationic Zinc Nanoparticles (CHZNPs) highlights the key costs involved in the synthesis process, including the raw materials such as zinc nitrate, chlorohexidine, and ionic liquids, as well as the solvents used. Preliminary estimates suggest that the cost of materials like zinc nitrate and chlorohexidine, along with the energy and labour required for nanoparticle synthesis, results in a cost-effective formulation for small-scale production. However, scaling-up production may incur additional costs due to equipment and operational requirements. Despite initial production costs being higher than conventional antibiotics, the cost effectiveness of CHZNPs is enhanced by their sustained release profile, which may reduce the need for frequent doses, ultimately leading to potential cost savings in treating antibiotic-resistant infections. Furthermore, the long-term economic benefits of utilising CHZNPs to combat resistant bacterial strains may outweigh initial development costs, especially when considering the potential market demand for such innovative therapies.

Zinc nanoparticles (ZNPs) offer several potential environmental benefits due to their biodegradability and relatively low toxicity compared to other metal nanoparticles. Zinc, being an essential trace element for many organisms, is naturally present in the environment, which may reduce concerns about long-term ecological impact. In addition, sustainable and green synthesis methods are being explored to minimise the environmental footprint of ZNPs. By carefully controlling the release and disposal of ZNPs, their environmental implications can be mitigated, making them a promising option for eco-friendly applications in drug delivery and other fields.

The successful translation of Chlorohexidine-loaded Cationic Zinc Nanoparticles (CHZNPs) into clinical applications presents promising therapeutic opportunities. However, this process involves addressing critical regulatory considerations, including rigorous testing for safety, efficacy, and biocompatibility to ensure the nanoparticles meet the required standards for human use. As nanomedicine advances, regulatory frameworks are evolving to facilitate the development of nanoparticle-based therapies. With thorough preclinical evaluations and collaboration with regulatory agencies, CHZNPs could provide a valuable addition to contemporary treatment options, especially in combating antibiotic-resistant infections.

In comparison to previous studies on cationic zinc nanoparticles (ZNPs) and chlorhexidine formulations, our results show significant improvements in antimicrobial efficacy. For example, studies by Smith et al. [61] and Lee et al. [62] demonstrated the antibacterial potential of ZNPs against *S. pneumoniae*, but the MIC values reported were relatively higher (100–200 μg/mL) compared to the MIC90 of 50 μg/mL observed in our CHZNPs formulation. Furthermore, while Johnson et al. [63] found that zinc nanoparticles alone exhibited limited sustained release, our CHZNPs formulation displayed a controlled release of chlorhexidine over 24 h, offering improved pharmacokinetic properties. These results suggest that the incorporation of chlorhexidine into zinc nanoparticle systems enhances their antimicrobial activity, making CHZNPs a promising alternative to conventional treatments for antibiotic-resistant bacterial infections.

## Conclusion

The CHX nanoformulation demonstrated superior efficacy in vitro, requiring lower amounts of CHX compared to commercially available CHX products. Our study demonstrates that CHX carrying CHZNPs nanoformulation possesses suitable physicochemical characteristics, as well as antibacterial properties. Collectively, our findings indicate that CHZNPs show promise as a potential alternative nanoformulation. Our results have shown significant positivity, which supports the advancement of future pharmacological investigations to determine the mechanisms of action of the nanostructured formulation. In addition, these results are encouraging for evaluating various concentrations of CHX in different nanoformulations, as well as assessing the penetration mechanisms and potential toxicity of these nanoformulations.

## Data Availability

The datasets analysed during the current study are available from the corresponding author on reasonable request.
